# Estimating cancer risk in carriers of Lynch syndrome variants in UK Biobank

**DOI:** 10.1136/jmg-2023-109791

**Published:** 2024-07-14

**Authors:** Eilidh Fummey, Pau Navarro, John-Paul Plazzer, Ian M Frayling, Sara Knott, Albert Tenesa

**Affiliations:** 1 MRC Human Genetics Unit, Institute of Genetics and Cancer, University of Edinburgh, Edinburgh, UK; 2 The Roslin Institute, University of Edinburgh, Roslin, Midlothian, UK; 3 Colorectal Medicine and Genetics, The Royal Melbourne Hospital, Parkville, Victoria, Australia; 4 The Centre for Familial Intestinal Cancer, St Mark's the National Bowel Hospital and Academic Institute, London, UK; 5 Institute of Cancer & Genetics, Cardiff University, Cardiff, UK; 6 Institute of Ecology and Evolution, University of Edinburgh, Edinburgh, UK

**Keywords:** Genetic Predisposition to Disease, Human Genetics, Neoplasms, Whole Exome Sequencing, Germ-Line Mutation

## Abstract

Lynch syndrome (LS) is an inherited cancer predisposition syndrome caused by genetic variants affecting DNA mismatch repair (MMR) genes *MLH1*, *MSH2*, *MSH6* and *PMS2*. Cancer risk in LS is estimated from cohorts of individuals ascertained by individual or family history of cancer, which may upwardly bias estimates.

**Methods:**

830 carriers of pathogenic or likely pathogenic (*path_MMR*) MMR gene variants classified by InSiGHT were identified in 454 756 UK Biobank (UKB) participants using whole-exome sequence. Nelson-Aalen survival analysis was used to estimate cumulative incidence of colorectal, endometrial and breast cancer (BC).

**Results:**

Cumulative incidence of colorectal and endometrial cancer (EC) by age 70 years was elevated in *path_MMR* carriers compared with non-carriers (colorectal: 11.8% (95% confidence interval (CI): 9.5% to 14.6%) vs 1.7% (95% CI: 1.6% to 1.7%), endometrial: 13.4% (95% CI: 10.2% to 17.6%) vs 1.0% (95% CI: 0.9% to 1.0%)), but the magnitude of this increase differed between genes. Cumulative BC incidence by age 70 years was not elevated in *path_MMR* carriers compared with non-carriers (8.9% (95% CI: 6.3% to 12.4%) vs 7.5% (95% CI: 7.4% to 7.6%)). Cumulative cancer incidence estimates in UKB were similar to estimates from the Prospective Lynch Syndrome Database for all genes and cancers, except there was no evidence for elevated EC risk in carriers of pathogenic *PMS2* variants in UKB.

**Conclusion:**

These results support offering incidentally identified carriers of any *path_MMR* surveillance to manage colorectal cancer risk. Incidentally identified carriers of pathogenic variants in *MLH1*, *MSH2* and *MSH6* would also benefit from interventions to reduce EC risk. The results suggest that BC is not an LS-related cancer.

**WHAT IS ALREADY KNOWN ON THIS TOPIC**

- Lynch syndrome (LS) is one of the most common genetic tumour syndromes and is associated with elevated colorectal and endometrial cancer (EC) risk that can be managed with increased surveillance and prophylactic surgery.
- An individual may receive a diagnosis of LS as an incidental finding if sequenced for diagnostic or research purposes.

**WHAT THIS STUDY ADDS**

- This study uses a large population-based cohort, which is not ascertained through individual or family history like disease cohorts, to characterise the cancer risk associated with LS gene variants.
- Risk estimates for colorectal, endometrial and breast cancer obtained from pathogenic LS gene variant carriers in this population-based cohort were concordant with published estimates from cohorts of individuals with an LS diagnosis undergoing cancer surveillance, with the exception of EC risk in carriers of pathogenic *PMS2* variants, which was not elevated relative to non-carriers.

**HOW THIS STUDY MIGHT AFFECT RESEARCH, PRACTICE OR POLICY**

- This suggests that individuals who receive an incidental diagnosis of LS would benefit from interventions to manage their colorectal and EC risk; however, this may not be required for EC risk in carriers of pathogenic *PMS2* variants.

## Introduction

Lynch syndrome (LS) is an autosomal dominant cancer predisposition syndrome caused by variants affecting one of four DNA mismatch repair (MMR) genes: *MLH1*, *MSH2*, *MSH6* or *PMS2*.^1^ Individuals with LS generally have increased risk of developing early-onset colorectal cancer (CRC) and some extracolonic cancers, such as endometrial cancer (EC). Understanding the cancer risk associated with likely pathogenic or pathogenic variants affecting MMR gene function (jointly referred to as *path_MMR*) is important for informing management plans for prevention and early detection of LS-associated cancers.

The majority of studies that estimate the cancer risk associated with *path_MMR* use cohorts of individuals with a diagnosis of LS. These cohorts are subject to ascertainment bias because only individuals who have been diagnosed, or have a close family member who was diagnosed, with an LS-related cancer are likely to receive mutational screening. In other hereditary cancer predisposition syndromes, this has led to overestimation of cancer risk.^2 3^

If the cancer risk associated with *path_MMR* is overestimated, this poses a problem for counselling individuals with incidentally identified *path_MMR*. The American College of Medical Genetics and Genomics and Genomics England recommend reporting incidentally discovered *path_MMR* to patients even if they are asymptomatic and have no family history suggestive of LS.^4 5^ Incidental identification of pathogenic variants is becoming more common because of large-scale biobanks that return genetic results to patients, such as the National Institute of Health’s All of Us research programme, and the increasing use of sequencing as a diagnostic tool in clinical genetics.^6 7^

Large population-based studies with sequencing data present an opportunity to estimate the penetrance of monogenic disease-causing variants without the bias introduced by selecting individuals based on disease status or family history.^8^ However, these studies are potentially subject to healthy volunteer bias and survivor bias, depending on the disease and study design, and thus may underestimate penetrance. Hence, such cohorts may provide a lower bound penetrance estimate, whereas classical studies of individuals diagnosed with rare genetic disease provide an upper bound.

The International Society for Gastrointestinal Hereditary Tumours (InSiGHT) maintains the Colon Cancer Gene Variant Database.^9^ This database was created to bring together new and existing data on the clinical significance of germline variants for CRC risk. An expert panel reviews this evidence and classifies variants as pathogenic or benign following the International Agency for Research on Cancer’s five-tier classification system.^10^

Here, carriers of MMR gene variants listed in the InSiGHT Colon Cancer Gene Variant Database (referred to as the InSiGHT database) are identified in a large population-based study, UK Biobank (UKB), using whole-exome sequence (WES) from 454 756 individuals. Carriers of *path_MMR* are used to generate cumulative incidence estimates for colorectal and EC, the most commonly diagnosed cancers in individuals with LS,^11^ and breast cancer (BC), given the mixed evidence surrounding whether BC risk is elevated in LS.^12–17^ The effect of sex and polygenic risk on CRC risk was investigated. Additionally, cancer risk associated with variants of uncertain significance (VUS) is examined.

## Methods

### Phenotypic data

UKB consists of ~500 000 individuals living in the UK, recruited between ages 40 and 69 years.^18^ At recruitment, individuals answered a health and lifestyle questionnaire, provided biological samples and consented to this being linked to their electronic health records, including cancer and death registries.

The cancer registry data come from two sources: NHS Digital for England and Wales and the National Records of Scotland NHS Central Register. For England and Wales, the data included recorded cancer cases from 1971 onwards, and for Scotland, cases were recorded from 1957 onwards.

Only cancers that were diagnosed prior to the relevant cancer registry’s censor date were considered. The censor dates (as calculated by UKB^19^) were 29 February 2020 for England and Wales and 31 January 2021 for Scotland. Death registry records were used to identify the appropriate end of observation for each participant.

The following International Classification of Diseases (ICD) codes were used to identify relevant cancer diagnoses: ICD-9 153-154 and ICD-10 C18-20 for CRC; ICD-9 182 and ICD-10 C54 for EC; and ICD-9 174 and ICD-10 C50 for BC.

Family history of CRC was determined from recruitment questionnaire data. Participants were asked ‘has/did your (father/mother/brothers or sisters) ever suffered from any of the following illnesses?’, for which one of the responses was ‘bowel cancer’.

### Identification of carriers of InSiGHT classified variants

Exome sequencing protocols in UKB have been described previously.^20^ Genotypes supported by less than 10 reads or with a genotyping quality of less than 20 were set to missing. Sites were removed if more than 10% of genotypes were missing after these filters were applied, or if the site quality was less than 20. Variants located in exons 11–15 of *PMS2* were not considered due to this region’s homology with pseudogene *PMS2CL*.

Chromosome, base pair position, reference and alternative alleles for 1932 InSiGHT classified variants were obtained from ClinVar^21^ and used to identify carriers from the UKB exome sequence. Variants are described relative to coding reference sequences on GRCh38.p14 (NC_000007.14): NM_000249.4(*MLH1*), NM_000251.2(*MSH2*), NM_000179.2(*MSH6*) and NM_000535.6(*PMS2*).

### Estimation of cumulative cancer incidence

Cumulative cancer incidence was estimated using a modified form of Nelson-Aalen survival analysis as described in Dominguez-Valentin *et al*.^22^ Incidence rates were estimated for 5-year intervals from ages 25 to 74, assuming no cancers occurred prior to the age of 25. The incidence rate within an interval is the number of events observed divided by the total number of years individuals were observed for within the interval, multiplied by five (the interval length). The cumulative incidences are obtained from incidence rates using the Nelson-Aalen estimator. Differences between survival curves were tested using a Wilcoxon test where the proportional hazards assumption was not met and a log rank test otherwise.

### Polygenic Risk Score calculation

Polygenic risk scores (PRS) for CRC were calculated for 694 *path_MMR* carriers who both self-identified as white British and had genetic principal components indicating European ancestry. The score was calculated as the sum of alleles at 76 variants as in Schmit *et al*,^23^ weighted by the effect estimates of the variants. This PRS was chosen as its weights were calculated in samples independent of UKB. Genotypes used in PRS calculation were obtained from the genotyping array data available for UKB participants, described in Bycroft *et al*.^18^ Cox regression was used to test the effect of the PRS on CRC risk, with sex included as a covariate. Each gene was analysed individually.

## Results

### 465 variants classified by InSiGHT present in UKB

Among 454 756 UKB participants, 465 variants classified by InSiGHT had at least one alternative allele carrier. Of these 465 variants, 160 were classified as benign or likely benign; 186 were VUS and 119 were pathogenic or likely pathogenic (*path_MMR*).

The 119 different *path_MMR* variants were carried by 830 individuals (~1 in 550 individuals). No individual carried more than one *path_MMR* and all individuals were heterozygous. Stratified by gene, 431 (51.9%) carried a pathogenic or likely pathogenic variant in *PMS2* (*path_PMS2*), 290 (34.9%) in *MSH6* (*path_MSH6*); 62 in *MSH1* (*path_MLH1*) (7.5%) and 47 in *MSH2* (*path_MSH2*) (5.7%).

**Figure 1:**
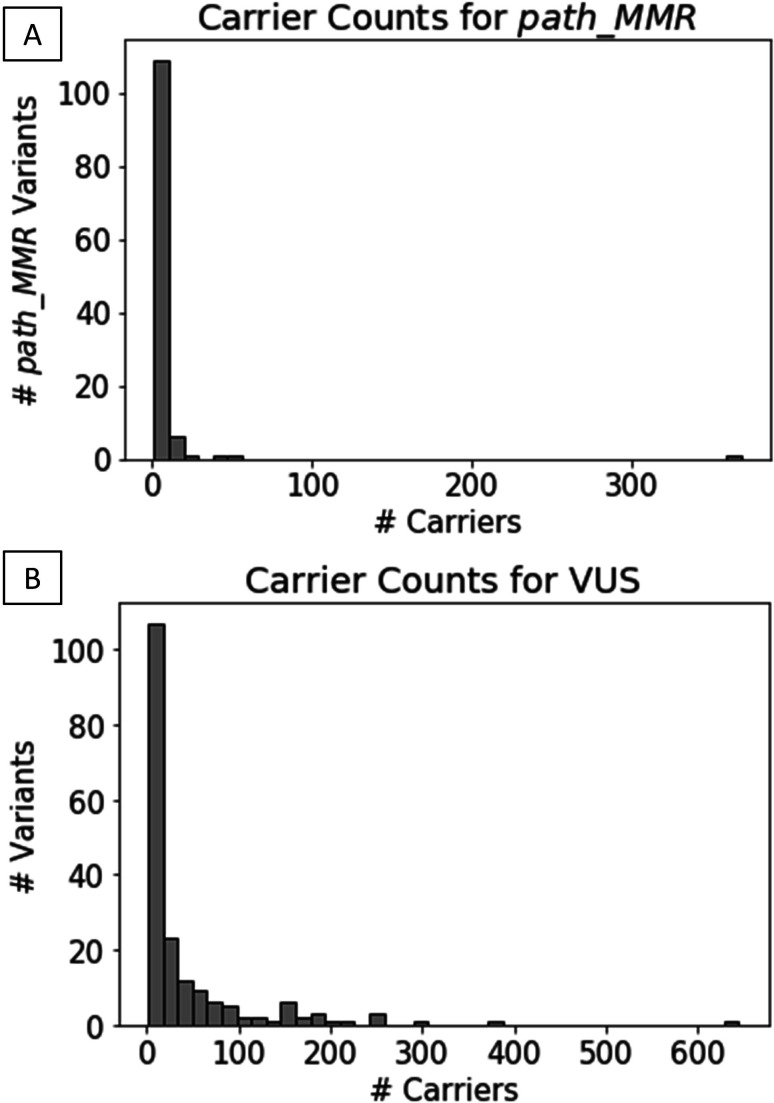
Histograms showing numbers of carriers in UK Biobank for mismatch repair (MMR) gene variants classified in the InSiGHT database as (A) pathogenic/likely pathogenic (*path_MMR*) and (B) of uncertain significance (VUS).

Of the 119 *path_MMR* represented, 52 (43.7%) were observed in a single UKB participant (figure 1A). All but one *path_MMR* (PMS2:c.137G>T, n=368) was observed in 50 or fewer participants. An additional 7849 UKB participants carried a VUS (approximately 1 in 60 individuals). The number of carriers per VUS ranged from 1 to 644 individuals (figure 1B).

### Elevated risk of colorectal and endometrial, but not breast, cancer in *path_MMR* carriers

In total, there were 7854 participants with diagnoses of CRC, 84 (1.1%) of which occurred in *path_MMR* carriers, and 2310 participants with diagnoses of EC, 54 (2.3%) of which occurred in *path_MMR* carriers. Nelson-Aalen curves for CRC and EC were fit for *path_MMR* carriers, stratified by gene, and for non-carriers. Leave-one-variant-out curves were generated for variants that made up more than 10% of carriers for a gene. These curves were created using individuals who carried *path_MMR* for a gene, excluding one particular variant, and were compared with the curve generated using all carriers of *path_MMR* for that gene to examine whether the shape of a curve for a gene was driven by a single variant. The leave-one-variant-out curves did not differ significantly from the all-variant curve: PMS2:c.137G>T (n=368; Wilcoxon tests: colorectal: W=0.05, p=0.82; endometrial: W=0.68, p=0.41), MSH6:c.3226C>T (n=50; log rank tests: colorectal: χ^2^=0.30, df=1, p=0.57; endometrial: χ^2^=0.47, df=1, p=0.49) and MSH6:c.3261dup (n=46; Wilcoxon test, colorectal: W=0.04, p=0.84; log rank test, endometrial: χ^2^=0.25, df=1, p=0.62). This suggested that the results are not driven by a single variant.

**Figure 2:**
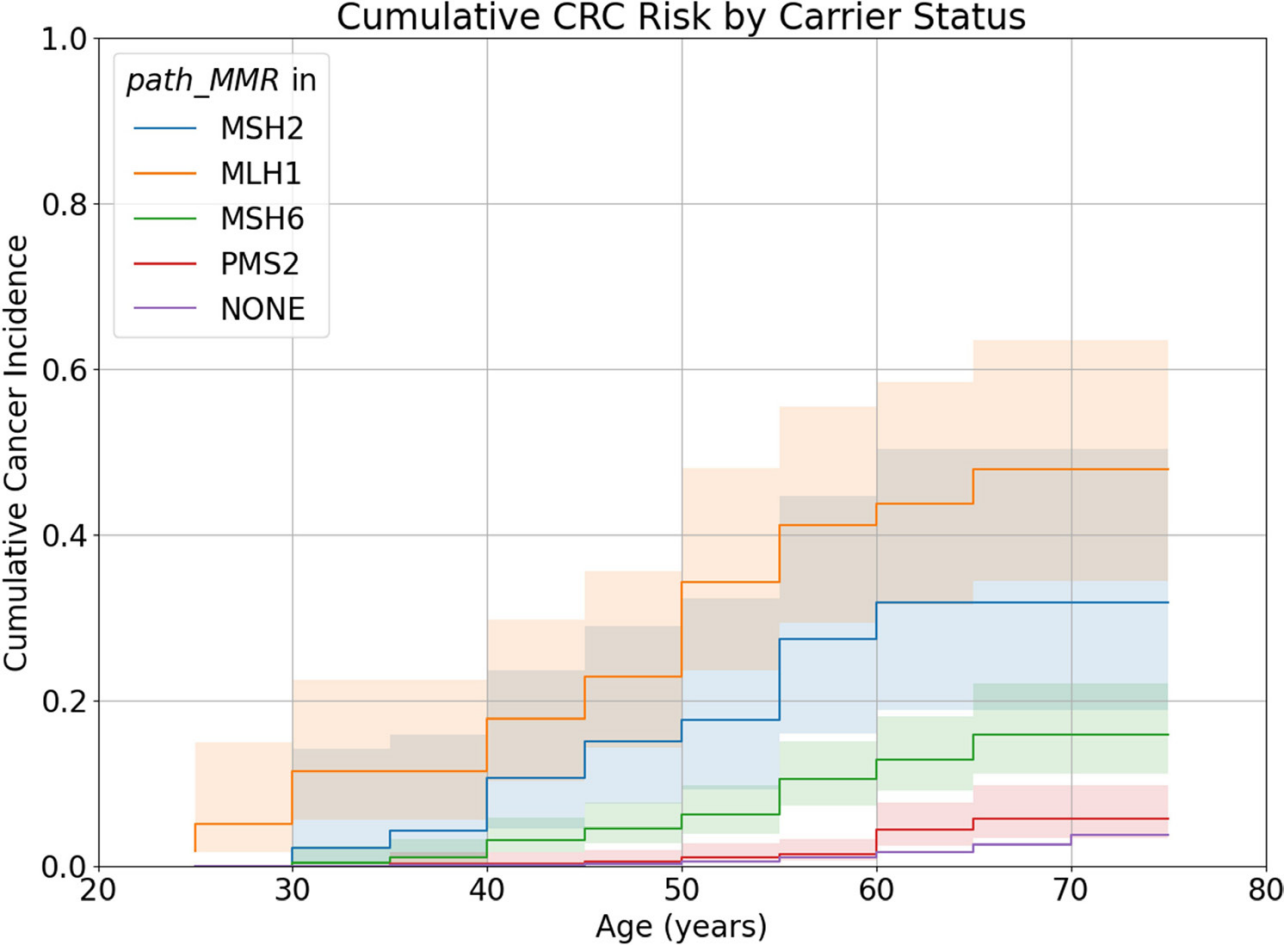
Nelson-Aalen curves of colorectal cancer for carriers of *path_MLH1*, *path_MSH2*, *path_MSH6*, *path_PMS2* and individuals who do not carry *path_MMR*. Shaded areas represent 95% CIs. CRC, colorectal cancer.

The CRC Nelson-Aalen curve for *path_MMR* carriers was significantly different to the curve for non-carriers (log rank test: χ^2^=398.45, df=1, p=1.20×10^−88^), with cumulative incidence of CRC by age 70 elevated in *path_MMR* carriers compared with non-carriers (11.8% (95% CI: 9.5% to 14.6%) vs 1.7% (95% CI: 1.6% to 1.7%)). Stratified by gene, *path_MMR* carriers had significantly different CRC curves from non-carriers at p<0.01 (log rank tests: *path_MLH1*: χ^2^=882.63, df=1, p=5.86×10^−194^; *path_MSH2*: χ^2^=236.81, df=1, p=1.92×10^−53^; *path_MSH6*: χ^2^=182.88, df=1, p=1.14×10^−41^; *path_PMS2*: χ^2^=7.05, df=1, p=0.008). In all 5-year intervals, cumulative incidence of CRC for *path_MMR* carriers in all genes was equal to or greater than that of non-carriers (figure 2). Among *path_MMR* carriers, the cumulative incidence of CRC was highest for *path_MLH1* carriers and lowest for *path_PMS2* carriers in all intervals. The median age of CRC diagnosis was 51.8 for *path_MLH1* carriers; 52.3 for *path_MSH2*; 59.5 for *path_MSH6*; 67.2 for *path_PMS2* and 65.2 for individuals who do not carry *path_MMR*.

**Figure 3:**
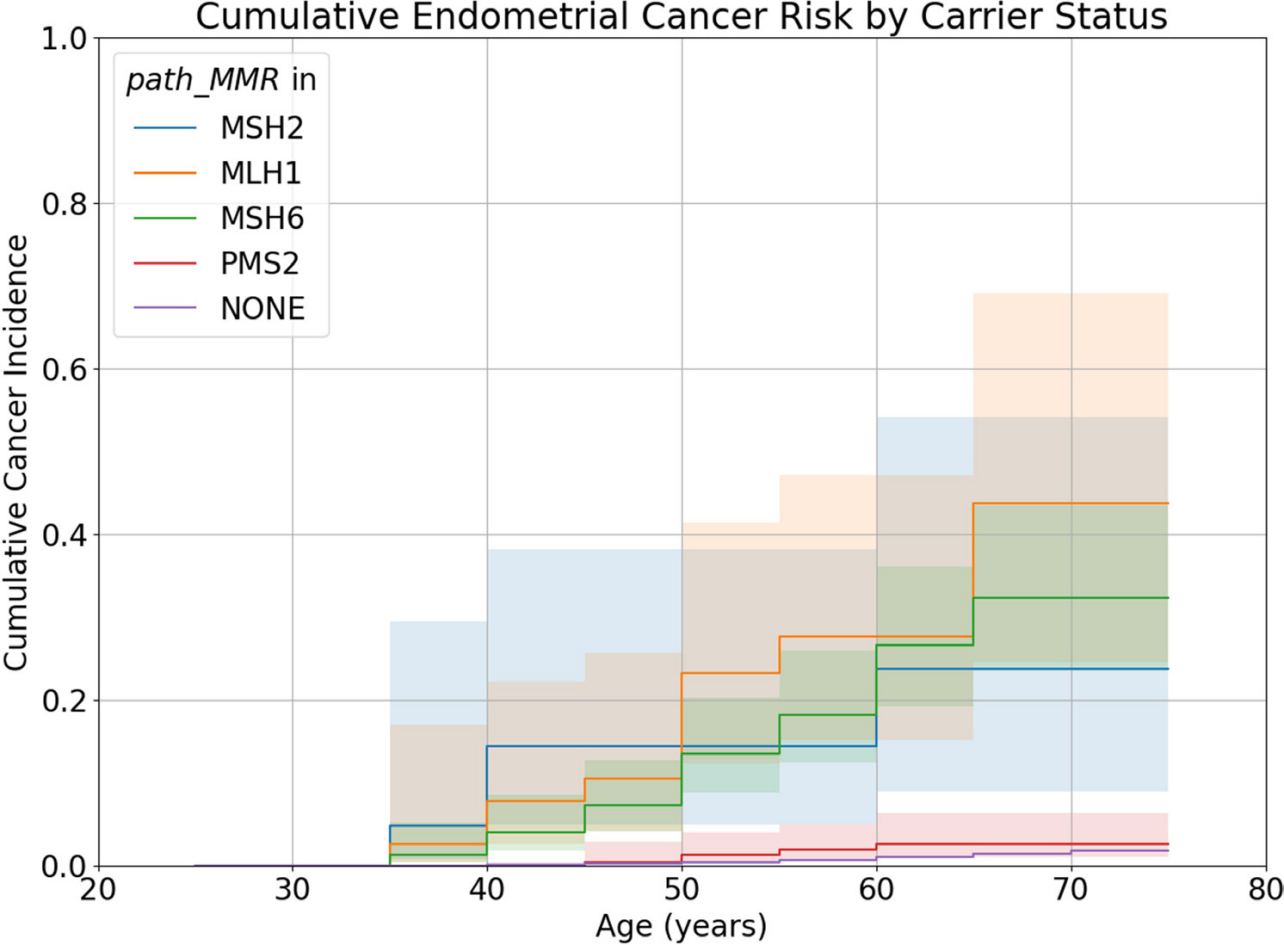
Nelson-Aalen curves of endometrial cancer for carriers of *path_MLH1*, *path_MSH2*, *path_MSH6*, *path_PMS2* and individuals who do not carry *path_MMR*. Shaded areas represent 95% CIs

The EC Nelson-Aalen curve for *path_MMR* carriers was also significantly different to the curve for non-carriers (log rank test: χ^2^=660.85, df=1, p=9.76×10^−146^), with cumulative incidence of EC by age 70 elevated in *path_MMR* carriers compared with non-carriers (13.4% (95% CI: 10.2% to 17.6%) vs 1.0% (95% CI: 0.9% to 1.0%)). When stratified by gene, only *path_MLH1*, *path_MSH2* and *path_MSH6* carriers had significantly different EC survival curves from non-carriers at p<0.01 (log rank tests: *path_MMR*: χ^2^=660.85, df=1, p=9.76×10^−146^; *path_MLH1*: χ^2^=470.93, df=1, p=2.01×10^−104^; *path_MSH2*: χ^2^=95.6, df=1, p=1.14×10^−22^; *path_MSH6*: χ^2^=935.13, df=1, p=2.27×10^−205^). In all 5-year intervals, the cumulative EC incidence was greater for carriers of *path_MMR* variants in these genes than it was for non-carriers (figure 3). The median age of onset for *path_MLH1*, *path_MSH2* and *path_MSH6* carriers was 56.4, 47.3 and 59.3, respectively, compared with 62.5 for non-carriers. However, *path_PMS2* carriers did not have a significantly different EC survival curve from non-carriers at p<0.01 (log rank test; χ^2^=3.58, df=1, p=0.059).

**Figure 4:**
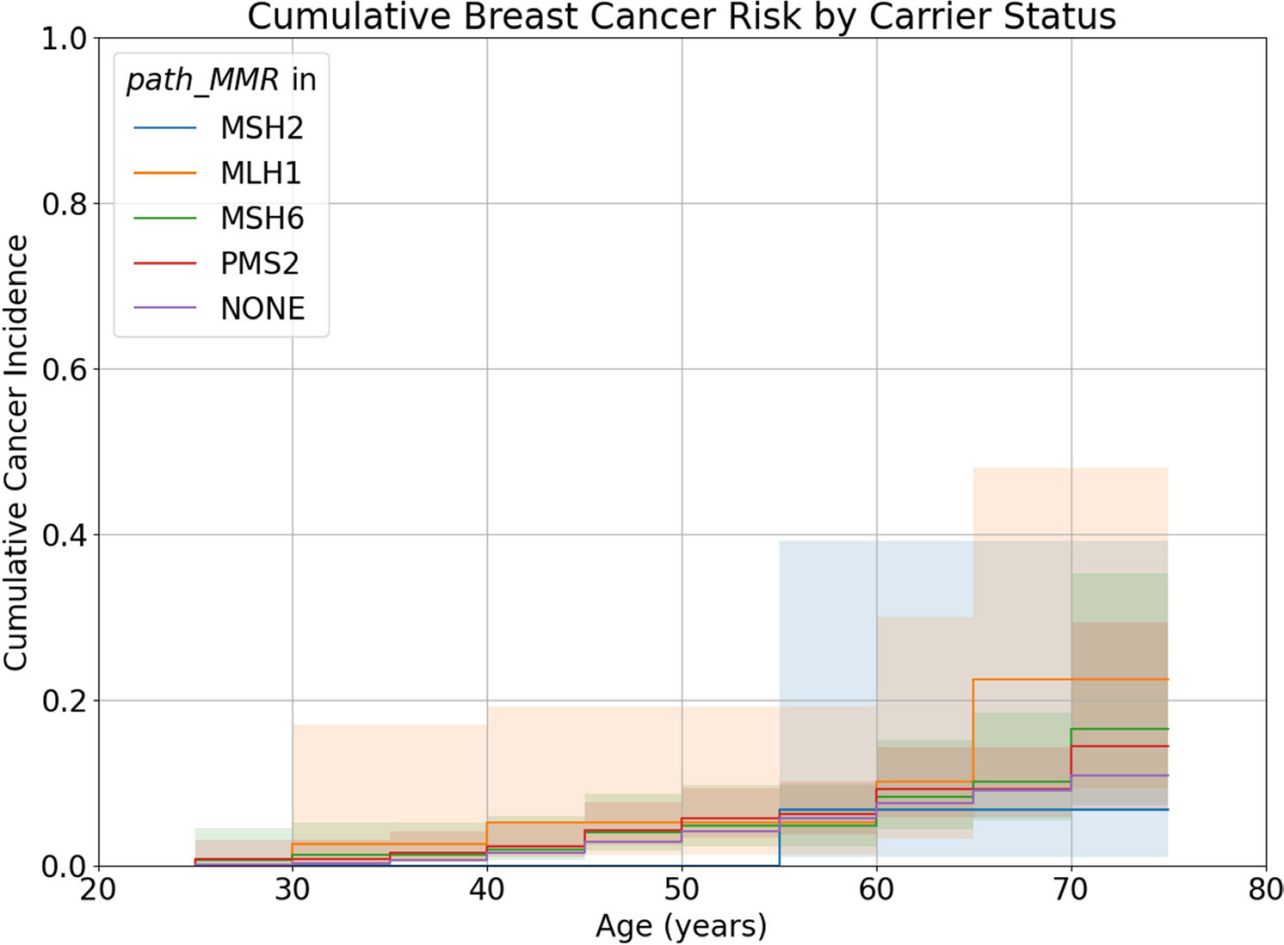
Nelson-Aalen curves of breast cancer for carriers of *path_MLH1*, *path_MSH2*, *path_MSH6*, *path_PMS2* and individuals who do not carry *path_MMR*. Shaded areas represent 95% CIs

*path_MMR* carrier Nelson-Aalen curves for BC were not significantly different to the curve for non-carriers (Wilcoxon tests: *path_MMR*: W=1.22, p=0.27; *path_MLH1*: W=0.21, p=0.31; *path_MSH2*: W=1.02, p=0.31; *path_MSH6*: W=0.12, p=0.73; *path_PMS2*: W=0.95, p=0.33) (figure 4). Cumulative incidence of BC by age 70 in carriers of *path_MMR* was similar to that of non-carriers (8.9% (95% CI: 6.3% to 12.4%) vs 7.5% (95% CI: 7.4% to 7.6%)).

### Sex differences in CRC risk observed for *path_MLH1* but not other *path_MMR*

**Figure 5:**
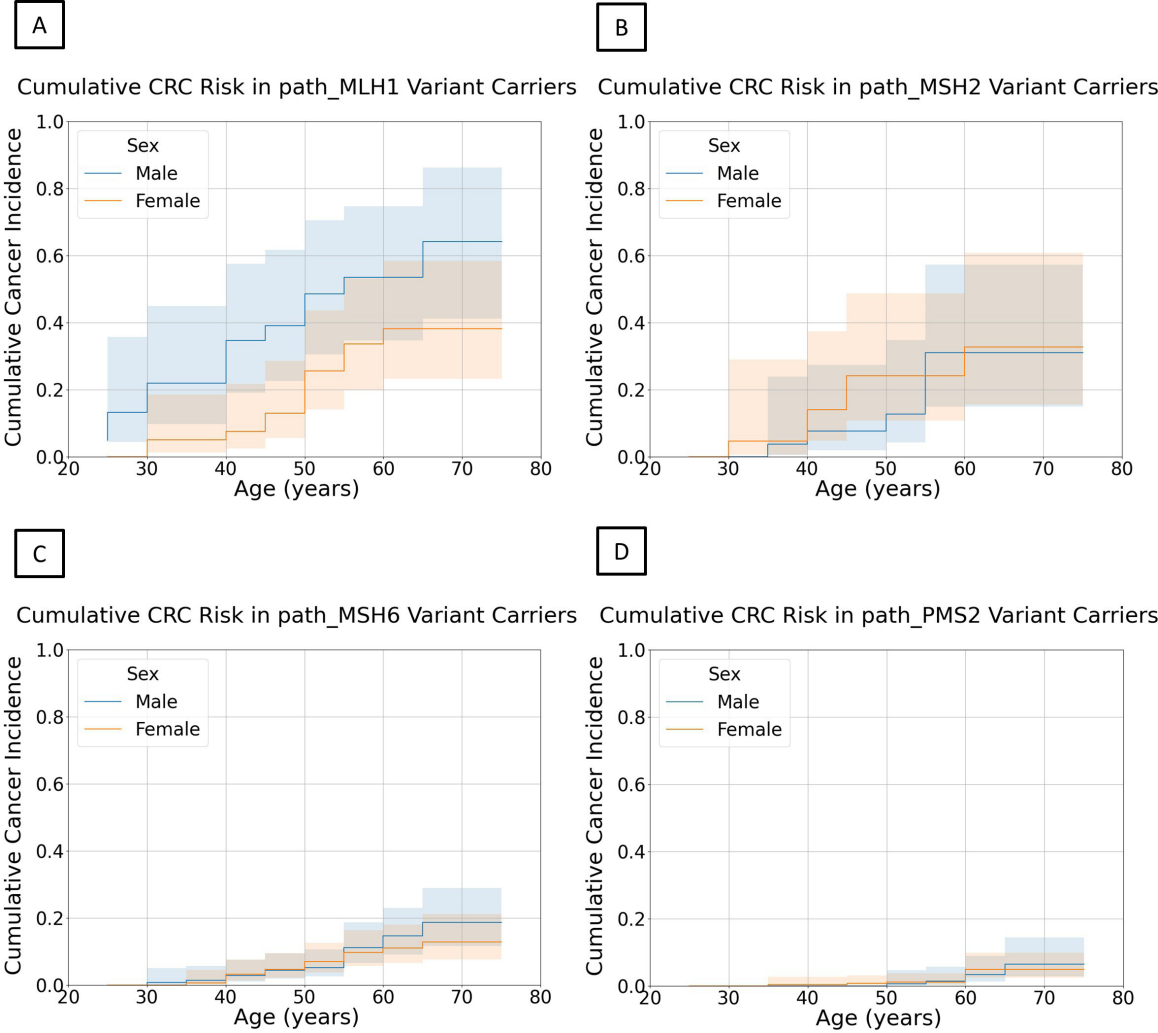
Nelson-Aalen curves of colorectal cancer for male and female carriers of (A) *path_MLH1*, (B) *path_MSH2*, (C) *path_MSH6*, and (D) *path_PMS2*. Shaded areas represent 95% CIs.

For each gene, CRC Nelson-Aalen curves of male and female *path_MMR* carriers were compared with establish whether sex influenced CRC risk (figure 5). The curve for male *path_MLH1* carriers was significantly different to the curve for female *path_MLH1* carriers at p<0.05 (logrank test: χ^2^=4.29, df=1, p=0.038), although the 95% CIs of both curves do intersect within all age intervals. Men had a higher cumulative incidence of CRC at age 70 (men: 53.5%, (95% CI: 34.7% to 74.7%); women: 38.2% (95% CI: 23.3% to 58.4%)) and an earlier median age of onset (men: 47.6; women: 55.1) (figure 5A). Differences between curves for men and women were not significant for any other gene (Wilcoxon tests: *path_MSH2*: W=0.44, p=0.50; *path_MSH6*: W=0.15, p=0.70; *path_PMS2*: W=0.12, p=0.73).

### *path_MMR* carriers report family history of CRC more frequently than non-carriers

Higher proportions of *path_MMR* carriers reported a close family history (parent or sibling) of CRC than non-carriers (24.1% vs 10.4%), but the proportion differs significantly between MMR genes (χ^2^ test: χ^2^=77.97, df=3, p=8.38×10^−17^). Over half of *path_MLH1* and *path_MSH2* carriers report a close family history of CRC (*path_MLH1*: 56.5%, *path_MSH2*: 51.1%). Reports of close family history of CRC were less frequent in *path_MSH6* carriers (27.2%) and even lower for *path_PMS2* carriers (14.4%). However, the proportion of close family history of CRC reported by *path_PMS2* carriers was still significantly greater than that of non-carriers (χ^2^ square test: χ^2^=6.76, df=1, p=0.0093).

### No evidence that polygenic risk modifies CRC risk in *path_MMR* carriers

Cox regression was used to test for the effect of the PRS developed in Schmit *et al* 23 on CRC risk within *path_MMR* carriers. White British UKB participants in the top 1% of this PRS had a fourfold increased risk of CRC compared with individuals in the bottom 1%. For *path_MSH2*, *path_MSH6* and *path_PMS2*, PRS was the sole predictor included in the Cox regression. For *path_MLH1*, the model included both sex and the PRS, as sex was a significant predictor at p<0.05 (Wald test: b=1.03, z=2.40, p=0.02). There is no evidence that the PRS modifies CRC risk for any gene (Wald test: *path_MLH1*: n=51, b=6.41, z=1.62, p=0.11; *path_MSH2*: n=35, b=1.47, z=0.28, p=0.78; *path_MSH6*: n=242, b=2.00, z=0.64, p=0.52; *path_PMS2*: n=366, b=3.98, z=0.95, p=0.34).

### Most VUS likely benign

**Figure 6:**
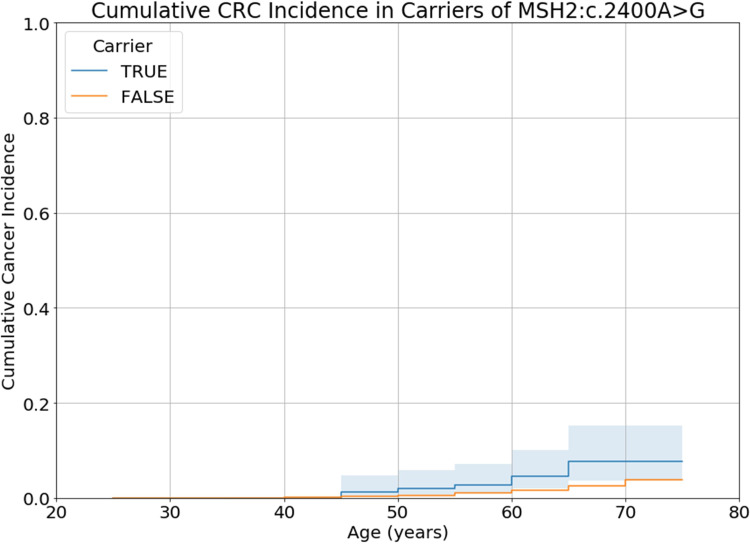
Nelson-Aalen curves of colorectal cancer for carriers (TRUE) of the variant of uncertain significance, MSH2:c.2400A>G, compared to non-carriers (FALSE). Shaded areas represent 95% CIs. CRC, colorectal cancer.

60 VUS were carried by 30 or more individuals. The proportion of CRC cases observed for carriers was greater than for non-carriers for 33 of these 60 VUS. None of the CRC Nelson-Aalen survival curves for these variants differed significantly from the non-carrier curve a p-value threshold, Bonferroni corrected for the number of VUS, of p<0.0008. One variant had a curve that was significant at a nominal significance threshold of p<0.05: MSH2:c.2400A>G (log rank test: χ^2^=8.80, df=1, p=0.003) (figure 6). The cumulative incidence of CRC by age 70 in carriers of MSH2:c.2400A>G was 7.6% (95% CI: 3.7% to 15.3%). This was lower than the cumulative incidence of CRC by age 70 for *path_MSH2* (31.8% (95% CI: 18.8% to 50.3%)) although it was greater than *path_PMS2* (4.3% (95% CI: 2.4% to 7.6%)). However, there was no evidence of increased family history of CRC in carriers of MSH2:c.2400A>G: 12.1% of carriers report a family history of CRC, compared with 10.5% of non-carriers (χ^2^ test; χ^2^=0.32, df=1, p=0.57). Moreover, MSH2:c.2400A>G is reported in ClinVar (accession VCV000090965.39) as a synonymous change (NP_000242.1:p.Leu800=) with no predicted impact on splicing and is annotated as likely benign by multiple other sources.

## Discussion

The analysis of carriers of InSiGHT classified *path_MMR* in UKB replicates known differences in the CRC and EC risk conferred by pathogenic variants in different MMR genes. EC risk is greater than CRC risk in *path_MSH6* carriers.^22 24^ CRC risk is less and generally later onset in *path_MSH6* carriers than *path_MLH1* and *path_MSH2* carriers.^22 24 25^ EC and CRC risk is notably less for *path_PMS2* carriers compared with carriers of *path_MMR* in other genes.^22^

In UKB, carriers of *path_MLH1* and *path_MSH2* are least frequently observed, many more *path_MSH6* carriers are identified, but the most carriers are observed for *path_PMS2*. This cannot be explained by greater numbers of InSiGHT classified *path_PMS2* variants compared with other genes, as there are more *path_MLH1* (535, 45.07%) and *path_MSH2* (454, 38.25%) variants than *path_MSH6* (167, 14.07%) or *path_PMS2* (31, 2.61%). Moreover, some regions of *PMS2* were not investigated due to pseudogene homology. The frequency of InSiGHT classified *path_MMR* in each gene in UKB is the inverse of the penetrance of these variants for CRC. This is expected if more penetrant alleles are subject to stronger purifying selection.^26 27^

In cohorts of individuals with an LS diagnosis, the opposite trend is typically observed.^24 27 28^ An example is Prospective Lynch Syndrome Database (PLSD), which collects data on carriers of *path_MMR* from the point of genetic diagnosis. At last publication, the database included 8500 carriers: *path_MLH1* (36.8%) and *path_MSH2* (37.3%) carriers are most common, followed by *path_MSH6* (19.4%), and the fewest carriers are observed for *path_PMS2* (6.5%).^22^

There are a number of potential explanations for this disparity between UKB and LS cohorts. First, there are differences in how *path_MMR* were identified. The short read sequencing technology used to sequence UKB participants cannot accurately genotype structural variants, such as whole-exon deletions or duplications, and thus these were not considered, although they are frequently identified in LS.^29^ Additionally, only variants classified by InSiGHT as pathogenic or likely pathogenic were considered as *path_MMR* to ensure sufficient weight of evidence for pathogenicity.

Cohort design may influence differences in *path_MMR* carrier frequency. If *path_MLH1* and *path_MSH2* carriers are more likely to be affected by cancer than *path_MSH6* and *path_PMS2* carriers prior to receiving an invitation to participate in UKB (which were sent out to individuals between the ages of 40 and 60), then they may be less likely to enrol.^30 31^ *path_MSH6* and *path_PMS2* carriers may be under-represented in LS cohorts because lower penetrance obscures the autosomal dominant inheritance pattern used to identify affected families.

Contrastingly, the distribution of carriers among genes in UKB is similar to the distribution of biallelic *path_MMR* carriers enrolled in the International Replication Repair Deficiency Consortium (*PMS2*: 61%, *MSH6*: 29%, *MSH2*: 5.5%, *MLH1*: 4.5%).^32^ This consortium enrols individuals with suspected constitutional mismatch repair deficiency syndrome (CMMRD): a highly penetrant cancer predisposition syndrome characterised by multiple childhood-onset cancers.^33^ If *path_PMS2* and *path_MSH6* are more common in the population, as the distribution in UKB suggests, this may explain why they are more often observed as a cause of CMMRD.

Despite differences in ascertainment between UKB and LS cohorts, CRC cumulative incidence estimates from UKB are similar to those in the literature.^15 22 25^ Using PLSD as an example, the cumulative CRC incidence by age 70 for male carriers of *path_MLH1* is 53.5% (95% CI: 34.7% to 74.7%) in UKB and 52.8% (95% CI: 46.1% to 59.8%) in PLSD; for *path_MSH2*, 31.0% (95% CI: 14.9% to 57.32%) vs 51.0% (95% CI: 42.8% to 59.7%); for *path_MSH6*, 14.6% (95% CI: 9.1% to 23.0%) vs 13.5% (95% CI: 7.1% to 24.8%) and for *path_PMS2* 3.4% (95% CI: 1.3% to 9.0%) vs 10.5% (95% CI: 2.7% to 36.0%) (online supplemental figure 1). As in UKB, female carriers of *path_MLH1* in PLSD have a lower cumulative CRC incidence by age 70 than male carriers: 42.1% (95% CI: 36.2% to 48.6%) in UKB vs 38.2% (95% CI: 23.25% to 58.39%) in PLSD. Values are also similar for female carriers of *path_MMR* in the other three genes: for *path_MSH2,* 32.8% (95% CI: 15.5% to 60.7%) in UKB vs 39.8% (95% CI: 33.5% to 46.7%) in PLSD; for *path_MSH6* 11.0% (95% CI: 6.6% to 18.1%) vs 17.3% (95% CI: 11.2% to 26.3%); and for *path_PMS2* 5.0% (95% CI: 2.5% to 9.9%) vs 8.5% (95% CI: 2.1% to 31.5%) (online supplemental figure 2). However, it is unclear if *path_MMR* carriers in UKB have been diagnosed with LS and are therefore receiving the CRC surveillance offered to individuals in PLSD.

10.1136/jmg-2023-109791.supp1 Supplementary data

The cumulative EC incidence by age 70 for *path_MLH1* is 43.7% (95% CI: 24.5% to 69.1%) in UKB vs 35.8% (95% CI: 29.9% to 42.5%) in PLSD; for *path_MSH2* 23.7% (95% CI: 8.95% to 54.1%) vs 42.1% (95% CI: 35.0% to 50.1%); for *path_MSH6* 32.4% (95% CI: 23.5% to 43.5%) vs 41.4% (95% CI: 32.3% to 52.0%); and for *path_PMS2* 2.6% (95% CI: 1.07% to 6.32%) vs 12.7% (95% CI: 5.5% to 27.9%) (figure 3). The only case where the 95% CI for UKB does not overlap the PLSD estimate and vice versa is for *path_PMS2*. In PLSD, carriers of *path_PMS2* have elevated EC risk but this trend is not observed in UKB. Note that 85% of female *path_PMS2* carriers in UKB carry c.137G>T, which has an allele frequency of 8.09×10^−4^ in UKB and 1.73×10^−4^ in gnomAD V.2.1.1.^34^ It is possible that c.137G>T is less penetrant than other *path_PMS2*, although the analysis of *path_PMS2* carriers excluding this variant also failed to find elevated EC risk. However, as there was a low number of individuals carrying *path_PMS2* that were not c.137G>T (n=39), there may have been insufficient power to detect an effect if penetrance is low. Further study is required to clarify the relationship between *path_PMS2* and EC risk.

Importantly, the similarity between UKB and PLSD does not suggest the existence of large subgroups of *path_MMR* carriers with low cancer risk that have been excluded from previous LS cancer risk estimates due to ascertainment bias. This is not the case in all monogenic diseases. One paper reported that the penetrance of diabetes in pathogenic *HNF1A*/*HNF4A* variant carriers was much lower in carriers identified through unselected cohorts (32%) vs cohorts of individuals with a molecular diagnosis (98%).^35^ However, there was no difference in diabetes penetrance between different ascertainment contexts for carriers of pathogenic *GCK* variants. The data presented here for LS suggest that variants in all four of the MMR genes discussed are more similar to *GCK* than *HNF1A*/*HNF4A*: the method of identification of variant carriers does not change their penetrance estimates to such an extent that it would warrant different clinical management of incidentally identified carriers. The only exception is that *path_PMS2* carriers are observed to have lower EC risk in UKB than previously reported.

Elevated incidence of BC has been reported in individuals carrying *path_MSH6* and *path_PMS2*.^12–14^ However, this is not consistently observed across all studies.^15–17^ Elevated BC incidence is more commonly observed in studies where *path_MMR* carriers are identified in cohorts of individuals referred for non-specific hereditary cancer screening. It has been argued that because individuals with *path_MMR* are typically identified through screening of CRC patients, families with elevated risk of BC may be excluded, leading to inconsistent findings.^13^

If this were the case, elevated BC incidence would be expected in *path_MSH6* and *path_PMS2* carriers in UKB. Rather, there is no evidence of increased BC risk in *path_MMR* carriers of any kind in UKB. Previous links between *path_MMR* and BC may be caused by higher rates of mammographic screening in individuals with a family history of LS-associated cancers, such as ovarian, leading to overdiagnosis.^15^ Additionally, due to their population frequencies, *path_MSH6* and *path_PMS2* may often be incidentally identified in individuals undergoing multigene hereditary cancer screening. The population undergoing this screening has a higher proportion of individuals with a BC diagnosis than the general population, so elevated BC risk in *path_MMR* carriers identified in this way could be attributed to ascertainment bias.^16^

One potential reason why one *path_MMR* carrier may develop CRC while another does not is additional genetic risk conferred by common variants. Previously, a 96 variant PRS was reported to be associated with CRC in UKB *path_MMR* carriers^36 37^; however, UKB samples made up 20% of the sample used to calculate the weights for this PRS, so there is a risk of overfitting.^38^ Studies that have tested the association between the 95 variant PRS and CRC in individuals with LS outside of UKB have failed to observe a significant relationship.^39 40^ Here, a 76 variant PRS for CRC was not associated with CRC risk in UKB *path_MMR* carriers. Although the power of this analysis is limited by small sample size, it appears likely that the relationship between this PRS and CRC risk is not strong enough for useful risk stratification in LS.

Around 1 in 60 UKB participants carries a variant classified as of uncertain significance in the InSiGHT database. VUS are commonly encountered when performing mutational testing of MMR genes, introducing uncertainty around diagnosis. For most VUS observed in UKB, there was little evidence of pathogenicity. Most VUS behaving like benign variants in UKB is consistent with the observation that VUS are more likely to be reclassified as benign or likely benign than upgraded to pathogenic or likely pathogenic.^41–43^ Cumulative incidences of CRC for VUS are provided in . This evidence can be incorporated into future discussions about reclassification of VUS in MMR genes.

In summary, these results do not advocate for the reclassification of any VUS as pathogenic. However, variants classified as pathogenic or likely pathogenic by InSiGHT are associated with increased risk of CRC in UKB, similar to published estimates. The same observation was made for EC risk in carriers of *path_MLH1*, *path_MSH2* and *path_MSH6*. This suggests that individuals incidentally found to carry these variants would benefit from interventions to manage these risks. However, the absence of a heightened risk of EC in carriers of *path_PMS2* necessitates further investigation into the appropriateness of interventions to manage EC risk in this subgroup. Lastly, failure to observe elevated BC risk in *path_MMR* carriers in UKB supports the stance that BC should not be considered an LS-related cancer.

## Data Availability

Data may be obtained from a third party and are not publicly available. Data used can be obtained through application to UK Biobank: https://www.ukbiobank.ac.uk/enable-your-research/apply-for-access.
