# Supplementary material for: Estimating cancer risk in carriers of Lynch syndrome variants in UK Biobank
Source: J Med Genet. 2024 Jul 14;61(9):861–9. doi: 10.1136/jmg-2023-109791 (PMC11420727; doi:10.1136/jmg-2023-109791)

Supplementary figures

Figure S1: Comparison between Nelson Aalen curves for colorectal cancer in UK Biobank (UKB, orange) and Prospective Lynch Syndrome Database (PLSD, blue) for male carriers of (A) *path_MLH1*, (B) *path_MSH2*, (C) *path_MSH6*, and (D) *path_PMS2*. Shaded areas represent 95% confidence intervals.

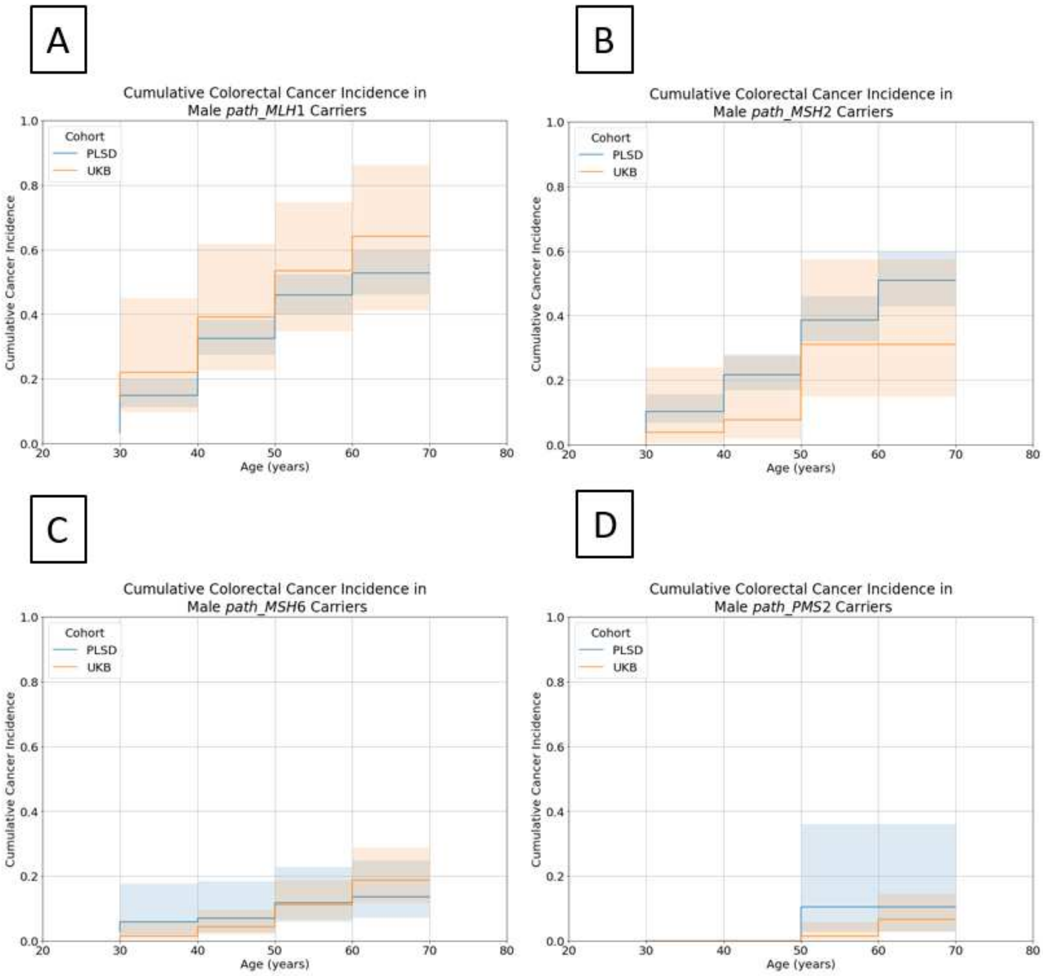

Figure S2: Comparison between Nelson Aalen curves for colorectal cancer in UK Biobank (UKB, orange) and Prospective Lynch Syndrome Database (PLSD, blue) for female carriers of (A) *path_MLH1*, (B) *path_MSH2*, (C) *path_MSH6*, and (D) *path_PMS2*. Shaded areas represent 95% confidence intervals.

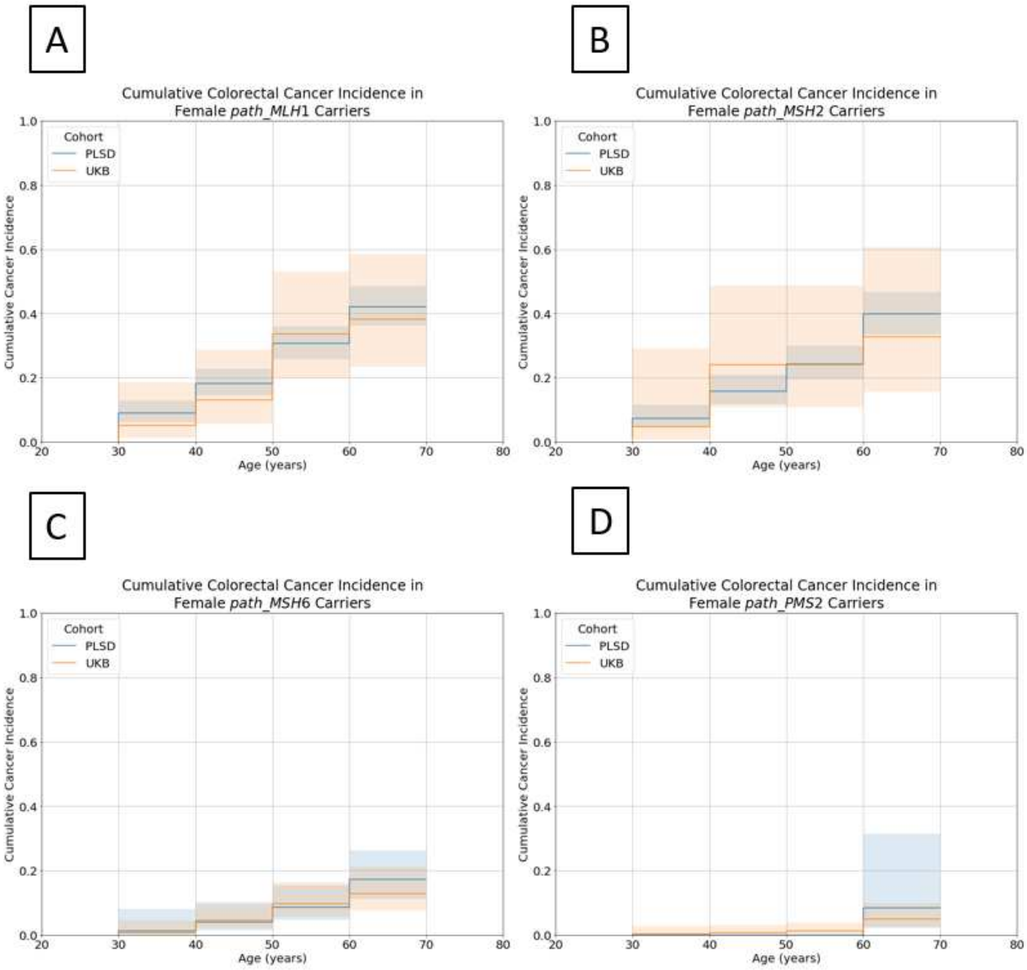

Figure S3: Comparison between Nelson Aalen curves for endometrial cancer in UK Biobank (UKB, orange) and Prospective Lynch Syndrome Database (PLSD, blue) for carriers of (A) *path_MLH1*, (B) *path_MSH2*, (C) *path_MSH6*, and (D) *path_PMS2*. Shaded areas represent 95% confidence intervals.

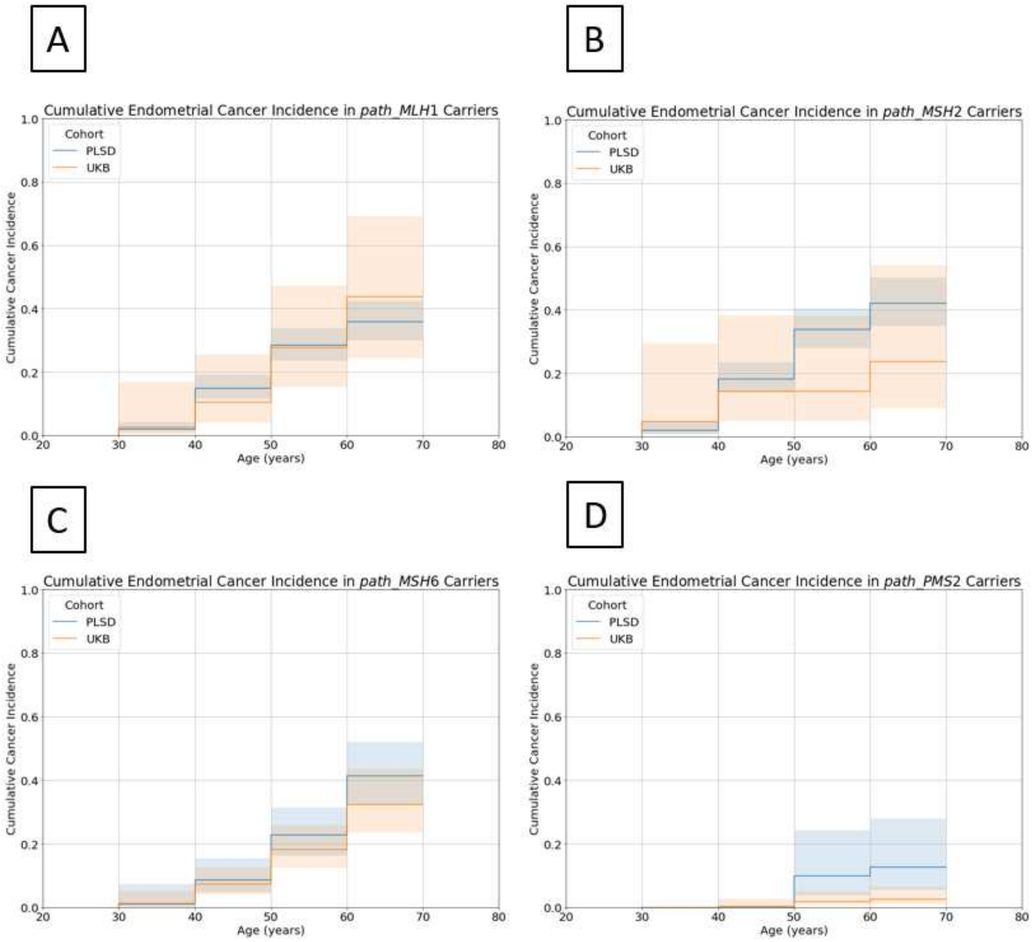

Supplement: Supplementary data [file jmg-2023-109791supp001.pdf]
